# Viable, Multi-Drug-Resistant Bacteria Recovered from E-Liquids Used with Commercial Electronic Cigarettes

**DOI:** 10.3390/ijerph22111725

**Published:** 2025-11-14

**Authors:** Suhana Chattopadhyay, Leena Malayil, Amy R. Sapkota

**Affiliations:** Department of Global, Environmental, and Occupational Health, University of Maryland School of Public Health, College Park, MD 20742, USA; lmalayil@umd.edu (L.M.); ars@umd.edu (A.R.S.)

**Keywords:** electronic cigarettes, electronic liquids, viable bacteria, antimicrobial resistance

## Abstract

The use of electronic cigarettes has increased in the U.S. with menthol and mint flavors showing notably higher sales. While research on the bacterial microbiome of traditional tobacco products is growing, particularly regarding menthol and nicotine effects, data regarding potential microbial contaminants within electronic liquids (e-liquids) remain limited. Additionally, the potential antibacterial properties of e-liquids remain sparse. To address these gaps, we evaluated the prevalence of viable bacteria in e-liquids; characterized their antimicrobial susceptibility patterns; and tested the antibacterial activity of the e-liquids. Two e-liquid flavors (menthol and non-menthol) across three different nicotine concentrations (0, 6 and 12 mg/mL) were tested using culture-based methods and Sanger sequencing. Antimicrobial susceptibility testing and e-liquid antibacterial activity assays were performed using the Kirby Bauer disc diffusion method. The majority of the isolates (63.15%) were identified as *Pseudomonas aeruginosa* and *Bacillus* spp. (*B. pumilus*, *B. megaterium* and *B. cereus*). Notably, *P. aeruginosa* and *P. fluorescens* isolates exhibited multidrug resistance against penicillin, tetracyclines, and phenicols. The e-liquids also demonstrated antimicrobial activity, inhibiting the growth of *B. cereus*, *P. aeruginosa*, and *Staphylococcus aureus*, with greater inhibition of *P. aeruginosa* growth at higher (12 mg/mL) compared to lower (0 mg/mL) nicotine concentrations across the menthol-flavored samples. These findings offer preliminary evidence of viable, multidrug-resistant bacteria and antibacterial properties in e-liquids, underscoring potential public health concerns regarding user exposure risks and microbial interactions, and emphasizing the need for continued surveillance of microbial safety in electronic cigarette products.

## 1. Introduction

Cigarette smoking rates have continued to decline over the last two decades in the United States (U.S.), from 20.9% in 2005 to 12.5% in 2020 [1,2]. However, more recently (2020–2022), total unit sales for electronic cigarettes (e-cigarettes) have increased by 57.3% in the U.S. [3]. Along with this rise, a 75.6% increase in total unit sales of non-tobacco flavored e-cigarette products (mint, menthol and other flavors) also occurred [3]. As a result, there is a significantly increasing number of e-cigarette users overall, and these individuals are being exposed to a mixture of chemicals, flavors and other potential contaminants present in e-cigarettes.

E-liquids (liquid used in e-cigarettes) typically contain stabilizing agents (propylene glycol/glycerol), nicotine and additives/flavors such as menthol, and are commonly available in a range of nicotine concentrations [4]. In addition to chemical constituents, e-cigarette users may also be exposed to bacterial contaminants originating from e-liquids. For example, a study identified bacterial endotoxin in 17 out of 75 tested e-liquid products [5]. Beyond e-cigarettes, a range of commercial tobacco products have been shown to contain diverse bacterial communities [6,7,8,9,10,11]. Across multiple studies, our group and others have demonstrated that the most common bacterial genera found in tobacco products (traditional cigarettes, little cigars, hookah and smokeless tobacco) are *Bacillus*, *Pseudomonas* and *Staphylococcus*, including some animal/human pathogenic species such as *B. pumilus*, *B. cereus*, *B. subtilis*, *S. epidermis*, *Anoxybacillus* and *Schlegella* [6,8,12,13,14,15,16,17,18,19]. Our work has further demonstrated that bacterial community composition is not only significantly affected by the brand and lots of tobacco products but also by the presence of mentholation and/or flavoring [17,18,19,20]. For example, mentholated commercial cigarettes are characterized by lower bacterial diversity compared to their non-mentholated counterparts [17,20], and nicotine concentrations can affect bacterial community composition in custom-made SPECTRUM cigarettes [21] indicating that the presence of menthol and varying nicotine levels may act to inhibit or change some types of tobacco-associated bacteria. Additionally, viable bacteria have been identified in mainstream cigarette smoke [22,23,24,25,26]. Furthermore, Fang et al. [27] recently reported that commercial cigarettes harbor potential human pathogens and antimicrobial resistance genes, underscoring another possible pathway for the spread of antimicrobial resistance.

Nevertheless, to our knowledge, no previous studies have evaluated the presence of viable bacteria in commercially available e-liquids, and there are very few data concerning the potential antibacterial activity that e-liquids may demonstrate against bacteria. Therefore, here we aimed to: (1) evaluate the prevalence of viable bacteria in commercially available e-liquids; (2) characterize antimicrobial susceptibility patterns among these bacteria; and (3) test whether or not the e-liquids themselves demonstrate antibacterial activity against bacterial types that are commonly detected in tobacco products.

## 2. Methods

### 2.1. Sample Procurement

One type of mentholated and one type of non-mentholated e-liquid product was procured from online stores: Menthol Tobacco by Kilo eLiquids Standard Series (M), and Smooth Tobacco by Kilo eLiquids Standard Series (non-menthol NM). For each product, three different nicotine concentrations were purchased (0 mg, 6 mg and 12 mg/mL) for a total of 6 different menthol/nicotine combinations: NM0, NM6, NM12, M0, M6 and M12.

### 2.2. Bacterial Detection and Sanger Sequencing

To isolate bacteria from each of the e-liquid types, 50 uL of each sample was spread-plated in triplicate on Trypticase Soy Agar (TSA) plates (Becton, Dickinson and Company, Franklin Lakes, NJ, USA). Plates were incubated at 37 °C for 24 h, and isolated colonies were picked, re-streaked and sub-cultured onto fresh TSA plates to obtain a pure culture. Single, purified colonies were then picked with sterile loops and streaked onto fresh TSA plates. Isolated colonies were then sent to Genewiz (South Plainfield, NJ, USA) for Sanger 16S rRNA sequencing. Resulting sequences in ‘fasta’ format were obtained from Genewiz and aligned against the 16S rRNA gene sequence database using the BLASTN 2.15.0 search tool.

### 2.3. Antimicrobial Susceptibility Testing

Each of the identified isolates was then subjected to antimicrobial susceptibility testing using the Kirby-Bauer disk-diffusion assay on Muller-Hinton Agar (BD Difco, Fairlakes, NJ, USA), according to the Clinical and Laboratory Standards Institute guidelines [28]. The tested antibiotics included five commonly used antibiotics: ampicillin (10 µg), tetracycline (30 µg), streptomycin (10 µg), penicillin (10 units) and chloramphenicol (30 µg). *Escherichia coli* ATCC 25922 was used as a positive control and molecular grade water was used as a negative control. Multidrug resistance (MDR) was defined as resistance to at least one antimicrobial agent across at least three antimicrobial classes [29].

### 2.4. Antibacterial Assays of E-Liquids Using the Agar Disk-Diffusion Technique

*Pseudomonas aeruginosa* (ATCC #27853), *Staphylococcus aureus* (ATCC #25293) and *Bacillus cereus* (ATCC #55055) were purchased from American Type Culture Collection (ATCC, Manassas, VA, USA). After proper resuscitation according to the manufacturer’s guidelines, the culture suspension concentrations were adjusted to a 0.5 MacFarland standard. Briefly, a colony was picked with a sterile loop and added to 2 mL of phosphate-buffered saline (PBS) to make a final concentration of ~1.5 × 10^8^ CFU/mL (0.5 MacFarland standard).

Fresh Mueller Hinton Agar (MHA) plates were spread plated using a sterile cotton swab with 10^8^ CFU/mL of each of the three ATCC culture strains in triplicate for each e-liquid concentration tested to achieve confluent growth. 6 mm diameter sterile filter disks (Whatman) were then placed on top of the ATCC culture strain with sterile forceps. 20 µL of three different concentrations of each e-liquid (no dilution, 1:10 dilution, and 1:100 dilution (with sterile water)) was pipetted onto each disk. For comparative purposes, tobacco was also obtained from Marlboro menthol and non-menthol traditional cigarettes and 0.5 g of dry tobacco was added to 5 mL of Luria–Bertani broth (LB, BD Difco, Fairlakes, NJ, USA) and vortexed to mix thoroughly. 20 µL of this solution was pipetted onto a sterile filter disk. For a negative control, 20 µL of sterile water was pipetted onto a disk. All plates were incubated for 24 h at 37 °C, and the diameter of the inhibition zone was then recorded in millimeters the next day. Statistical significance was tested using Kruskal–Wallis non-parametric ANOVA and a *p* value of < 0.05 was considered significant.

To understand whether the antibacterial properties of the e-liquids change over time, we repeated the disk diffusion assay after incubating the e-liquid samples at room temperature (20 °C and 50% relative humidity) for 7 days and 14 days.

## 3. Results

### 3.1. Isolate Identification

**Figure 1:**
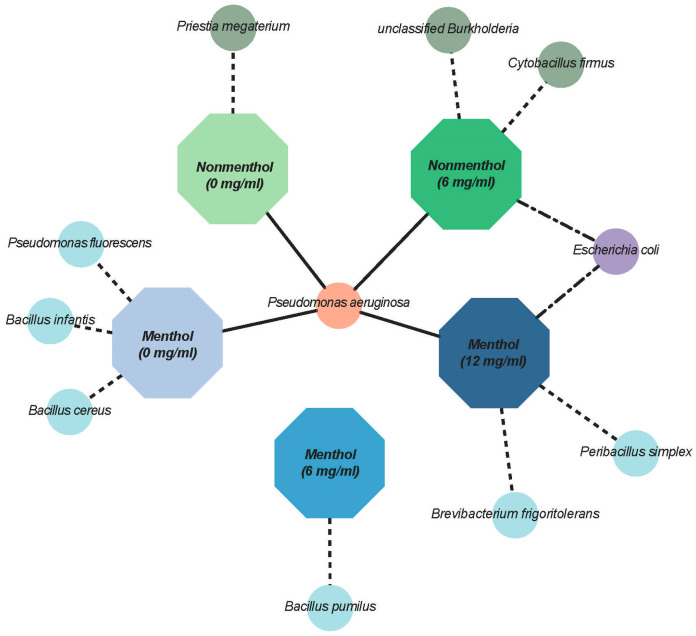
Network plot showing unique and shared bacterial species across the tested e-liquids. No isolates were recovered from non-menthol (12 mg/mL) e-liquids. Menthol samples are represented in blue and non-menthols in green octagons. Bacterial species are represented by ellipses, with unique species connected via dashed lines, shared species between two e-liquids via dot-dashed lines, and species shared between more than two e-liquids via solid lines.

Isolated colonies from all of the plates were critically assessed for phenotypic differences with the naked eye. Four phenotypically different colonies were identified, and two similar colonies for each phenotype were aseptically picked from each of the five (NM0, NM6, M0, M6, M12) different sample types for a total of 40 isolates. No colonies grew on the plate from the NM12 sample. Of the 40 isolates, 48% (19 isolates) were successfully identified using Sanger sequencing. Specifically, 3 isolates were recovered from NM0, 7 from NM6, 4 from M0, one from M6 and 4 from M12. The majority of the identified isolates included bacterial species belonging to *Pseudomonas aeruginosa* (42.1% of identified isolates) followed by *Bacillus* spp. (21%) (Figure 1). *P. aeruginosa* was found in NM0, NM6, M0 and M12 samples, while *P. fluorescens* were identified in the M0 sample only. While *E. coli* was identified in NM6 and M12, *Bacillus* spp. was only identified in the menthol samples (M0 and M6).

### 3.2. Antibiotic Resistance Profiles of Identified Isolates

All of the identified isolates were resistant to penicillin (Table 1), and all *P. aeruginosa* and *P. fluorescens* isolates were multidrug resistant to penicillin, tetracyclines, and phenicols. While six isolates (identified as *P. aeruginosa* and *Cytobacillus firmus*) recovered from the non-menthol flavored e-liquids were resistant against ampicillin, only one isolate (identified as *P. fluorescens*) recovered from the menthol flavored e-liquid was resistant (Table 1). Among the isolates recovered from non-menthol e-liquids, two (both identified as *P. aeruginosa*) were resistant against tetracycline and three (all identified as *P. aeruginosa*) were resistant to chloramphenicol.

### 3.3. Antibacterial Properties of the E-Liquids

**Figure 2:**
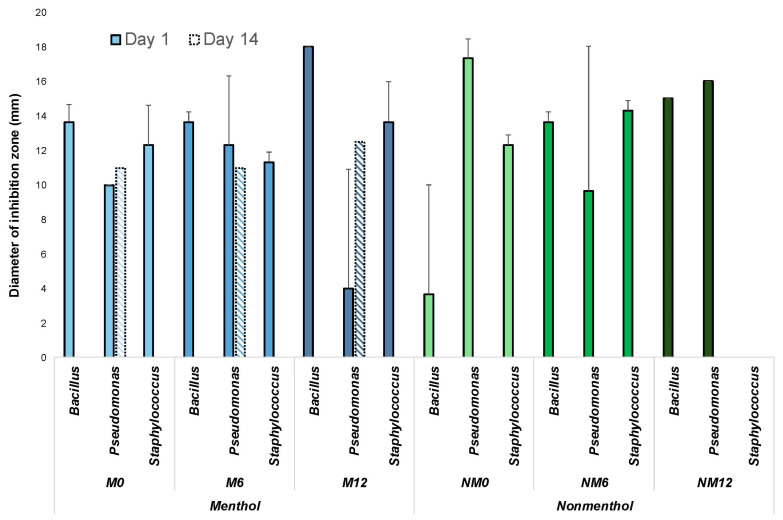
Antibacterial properties of non-diluted e-liquids against *B. cereus*, *P. aeruginosa* and *S. aureus* represented by the size of the inhibition zones. Larger inhibition zones indicate greater antibacterial activity against these bacterial isolates. Filled in bars indicate day 1 and patterned bars indicate day 14. Menthol flavors (M) are represented by blue bars and non-menthol flavors (NM) are represented by green bars. Menthol 0 mg/mL = M0, Menthol 6 mg/mL = M6, Menthol 12 mg/mL = M12, Non-menthol 0 mg/mL = NM0, Non-menthol 6 mg/mL = NM6, and Non-menthol 12 mg/mL = NM12. Lighter shades of blue/green represent lower nicotine concentrations, while darker shades of blue/green represent higher levels of nicotine.

For all three ATCC bacterial strains tested (*B. cereus*, *P. aeruginosa* and *S. aureus*), we observed zones of inhibition for all e-liquids and these zones of inhibition were the largest on day 1 when compared to that from day 14 (Figure 2). Comparing across nicotine levels among the menthol and non-menthol flavors tested, there was no significant difference (*p* < 0.05) in the diameters of inhibition zones between low (0 mg/mL), medium (6 mg/mL) and high (12 mg/mL) concentrations of nicotine content from the e-liquids. After the e-liquids were incubated at room temperature for 14 days, there were no measurable zones of inhibition for all non-menthol e-liquids (data for 7 days of incubation not shown in the figure). Across the menthol samples, while plates for *B. cereus* and *S. aureus* did not have any zones of inhibition, inhibition against *P. aeruginosa* growth was observed with slightly greater inhibition at higher concentrations of nicotine (M0: 11 mm, M12: 12.5 mm). In comparison, across both menthol and non-menthol cigarette samples, no zones of inhibition were observed, suggesting that cigarette tobacco did not possess antibacterial properties against the tested ATCC bacterial strains.

## 4. Discussion

In this study, we demonstrated for the first time that commercially available e-liquids can harbor not only viable bacteria but also antibiotic-resistant bacteria which could potentially be transferred to the smokers’ oral cavity. Previous studies have shown that exposure to e-liquid aerosols significantly increases biofilm formation by *Streptococcus mutans* and enhances *Staphylococcus aureus* attachment to oral epithelial cells [30,31]. These findings suggest that bacteria could not only survive aerosol exposure but also become more resilient, with an increased potential for colonization. Complementing these findings, our previous work demonstrated that tobacco-associated bacteria from cigarettes can survive aerosolization, are present in mainstream smoke [26] and may be transferred to the users’ oral cavities. These findings collectively suggest that e-cigarette, as well as cigarette, aerosols can support the survival and persistence of aerosolized bacteria, while simultaneously enhancing their virulence and biofilm-forming capacity within the oral cavity.

Besides identifying viable bacteria in e-liquids, our findings also revealed that all bacterial isolates from the e-liquids were resistant to penicillin but sensitive to streptomycin. In addition, the majority of the isolates derived from the mentholated samples were susceptible to all tested antibiotics. The detection of viable and antibiotic-resistant bacteria in e-liquids raises important concerns regarding the potential for oral and respiratory microbial dysbiosis among e-cigarette users. The oral cavity represents a critical gateway for microbial exchange, and disruption of its microbial balance has been linked to a range of inflammatory and systemic conditions, including periodontitis, cardiovascular disease, and respiratory infections [30,32,33]. Continuous exposure to aerosols containing viable bacteria or bacterial endotoxins could alter the composition and function of the oral microbiome, potentially favoring colonization by opportunistic or pathogenic species [34]. Furthermore, the presence of antibiotic-resistant bacteria in e-liquids may serve as a reservoir for horizontal gene transfer, facilitating the dissemination of resistance genes within oral microbial communities [35,36]. This risk could be amplified by the frequent and repeated nature of vaping, which provides both exposure and environmental conditions (humidity, temperature, nutrient residues) that may enhance bacterial persistence [37].

Of the multiple bacterial isolates that could be identified in our study, the majority were identified as *P. aeruginosa*. *P. aeruginosa* is an opportunistic pathogen that has been associated with chronic obstructive pulmonary disease (COPD) and ventilator-associated pneumonia (VAP) [38,39]. Additionally, it is also included as an ESKAPE pathogen (*Enterococcus faecium*, *Staphylococcus aureus*, *Klebsiella pneumoniae*, *Acinetobacter baumannii*, *Pseudomonas aeruginosa*, and *Enterobacter* species), a group of bacterial pathogens recognized by the World Health Organization as leading causes of nosocomial infections worldwide for which effective treatments are urgently needed [40]. Previous studies from our group and others have identified *Pseudomonas* routinely in multiple tobacco products including cigarettes, smokeless tobacco, little cigars, and hookah [8,14,19,20,41,42]. Specifically, *P. aeruginosa* has also been detected in cigarettes, smokeless tobacco and hookah [6,7,22,43,44]. Although multiple studies have detected *P. aeruginosa* in tobacco products, few have isolated the bacterium using culture-based methods, and to our knowledge, none have characterized the antibiotic resistance profiles of tobacco-related *P. aeruginosa*. In our study, while a few of the identified *Pseudomonas aeruginosa* isolates were resistant to one individual antibiotic, several isolates were multi-drug resistant (resistant to three different classes of antibiotics).

In addition to *P. aeruginosa*, the next major species identified in the e-liquids belonged to the *Bacillus* genus and included *B. megaterium, B. cereus, B. infantis*, and *B. pumilus*. *Bacillus* spp. is known to form endospores which enable the bacteria to be highly resistant to environmental stresses including heat [45,46]. Our group has previously detected *Bacillus* sp. in diverse commercial tobacco products [9,17,18,19,20], as well as the enriched smoke extract of traditional cigarettes [26]. Several of the *Bacillus* spp. detected, including *B. pumilus*, *B. megaterium* and *B. cereus*, are known to cause animal/human diseases ranging from foodborne illness to systemic diseases such as septicemia and meningitis [47,48].

These findings underscore the potential for vaping products to act as overlooked sources of environmental pathogens, particularly for individuals with preexisting respiratory or immune-compromised conditions [49,50]. Inhalation of contaminated aerosols could expose deeper regions of the respiratory tract to viable bacteria or bacterial endotoxins, potentially exacerbating pulmonary inflammation or infection risk [51]. In addition, since many e-liquids are stored at room temperature and repeatedly opened, they may provide favorable conditions for microbial proliferation and biofilm formation on device components [52]. The identification of antibiotic-resistant strains such as *P. aeruginosa* further emphasizes the need for stricter microbial quality control measures during the production, storage, and regulation of e-liquids [53].

Given our focus on bacterial contaminants, a comparative look at antimicrobial-resistance profiles indicates both overlaps and important differences between e-liquids and traditional tobacco products. Studies of commercially available e-liquids and e-cigarette vapors show that exposure to e-liquid aerosols can induce biofilm formation, greater tolerance to host antimicrobial peptides, and phenotypes associated with increased antibiotic tolerance in organisms such as *S. aureus* [54,55]. In contrast, conventional tobacco products and cigarette waste appear to serve as broader environmental reservoirs of antibiotic-resistance genes and compounds in smoke and waste that promote horizontal transfer and selection of resistance determinants across diverse bacterial taxa [27,56].

From the tested e-liquids, we recovered fewer isolates from the menthol-flavored e-liquids when compared to the non-menthol counterparts, which might be attributed to the antibacterial properties of menthol [57], as well as the antibacterial properties of propylene glycol (PG) and vegetable glycerin (VG) [54]. While most manufacturers of e-liquids do not reveal the constituents of the products, they usually consist of water, varying concentrations of a PG and VG base (ratios ranging from 30–70%), added nicotine and flavors, as well as solvents such as ethanol. PG and VG are both considered antibacterial and bacteriostatic, although they are not classified as bactericidal [58]. Previous studies on the bactericidal activities of PG and VG have shown partial effectiveness (25–50%) against *Enterococcus faecalis*, *E*. *coli* and *Streptococcus mutans* to 100% effectiveness against *S. aureus* [59]. Our results also demonstrated effective inhibition against *B. cereus*, *P. aeruginosa*, and *S. aureus* for all tested e-liquids except for non-menthol (12 mg/mL) e-liquid samples against *S. aureus*. This result was similar to a previous study that demonstrated antibacterial activity of commercial e-liquids against several ATCC bacterial strains, including *P. aeruginosa* and *S. aureus* [54]. While this could be due to the presence of PG in the e-liquid, it could also be attributed to other unknown ingredients in the product. Interestingly, none of the isolates that we recovered from the e-liquids were identified as *S. aureus*, so perhaps the e-liquid constituents prevent the growth or persistence of this microorganism in these products. Nevertheless, in previous studies, tobacco smoke and nicotine treatments have been shown to increase biofilm formation for *S. aureus* while suppressing its virulence [60].

Previous studies using dose-dependent bacterial growth inhibition techniques have demonstrated a reduction in bacterial colony forming units associated with nicotine, with >50% inhibition occurring at nicotine concentrations ranging from 100 to 250 µg/mL [61]. Yet, an important finding from our study was that there was no significant difference in the number of colonies recovered between the different concentrations of nicotine for each of the tested e-liquids. This might be because our colonies were recovered from non-diluted e-liquids (which contain potentially antibacterial constituents) while the above-mentioned study tested bacterial growth in commercially available purified nicotine.

As in any laboratory-based study, this study had several limitations. First, we used a non-selective culture medium (TSA) under aerobic conditions to isolate potentially viable bacteria present in e-liquids. While this approach provided valuable preliminary evidence of viable bacteria within e-liquids, it limited the diversity of bacteria that could be recovered. Future studies could employ selective media to target and recover bacteria of higher diversity. Second, we included a single brand of commercial e-liquids. Since previous studies have demonstrated differences in bacterial diversity between different brands of tobacco products [9,17,18,19], future studies should include a variety of e-liquids brands available on the market. Finally, since it is estimated that only less than 2% of bacteria in the environment are culturable [62], it is necessary to employ culture-independent techniques, such as metagenomic sequencing, to comprehensively characterize the bacterial microbiota of e-liquids in future studies.

In conclusion, we demonstrated the presence of viable, multi-drug-resistant bacteria in commercially available e-liquids. On the other hand, we also showed that some e-liquid solutions are antibacterial and could suppress bacterial growth. Given our findings, further investigations concerning the presence of microbial contaminants across diverse brands of e-liquids are warranted. Moreover, further studies are necessary to evaluate the potential survival of these bacterial species in the e-liquids over time, as well as the potential transfer of these microorganisms to the users’ oral cavity through vaping. Finally, public health guidelines that currently focus primarily on chemical toxicity of smoked products may need to be expanded to include microbial safety standards. Understanding how e-liquid composition, flavor additives, and storage duration influence microbial survival could inform safer product design and reduce the risk of user exposure to harmful microorganisms [63].

## Figures and Tables

**Table 1:** Antimicrobial susceptibility profiles of isolates recovered from e-liquids against ampicillin, tetracycline, streptomycin, penicillin, and chloramphenicol. Red indicates resistance (R), yellow indicates intermediate susceptibility (I), and green indicates susceptibility (S), based on CLSI breakpoints guidelines for bacterial pathogens.

E-Liquid	Bacteria	Ampicillin	Tetracycline	Streptomycin	Penicillin	Chloramphenicol
Menthol (0 mg/mL)	*Pseudomonas aeruginosa*	I	S	S	R	S
	*Bacillus cereus*	I	S	S	R	S
	*Pseudomonas fluorescens*	R	I	S	R	I
	*Bacillus infantis*	S	S	S	R	S
Menthol (6 mg/mL)	*Bacillus pumilus*	S	S	S	R	S
Menthol (12 mg/mL)	*Peribacillus simplex*	S	S	S	R	S
	*Pseudomonas aeruginosa*	S	S	S	R	S
	*Escherichia coli*	S	S	S	R	S
	*[Brevibacterium] frigoritolerans*	S	S	S	R	S
Non-menthol (0 mg/mL)	*Pseudomonas aeruginosa*	R	R	S	R	R
	*Priestia megaterium*	S	S	S	R	S
	*Pseudomonas aeruginosa*	R	I	S	R	I
Non-menthol (6 mg/mL)	*Pseudomonas aeruginosa*	S	S	S	R	S
	*Escherichia coli*	S	S	S	R	S
	*Cytobacillus firmus*	R	S	S	R	S
	*Pseudomonas aeruginosa*	R	S	S	R	S
	*unclassified Burkholderia*	S	S	S	R	S
	*Pseudomonas aeruginosa*	R	R	S	R	R
	*Pseudomonas aeruginosa*	R	I	S	R	R

## Data Availability

All data related to this work are presented in the manuscript.
